# New nonbinary code bounds based on divisibility arguments

**DOI:** 10.1007/s10623-017-0366-0

**Published:** 2017-05-20

**Authors:** Sven C. Polak

**Affiliations:** Korteweg-De Vries Institute for Mathematics, University of Amsterdam, Amsterdam, The Netherlands

**Keywords:** Code, Nonbinary code, Upper bounds, Kirkman system, Divisibility, Symmetric net, 94B65, 05B30

## Abstract

For $q,n,d \in \mathbb {N}$, let $A_q(n,d)$ be the maximum size of a code $C \subseteq [q]^n$ with minimum distance at least *d*. We give a divisibility argument resulting in the new upper bounds $A_5(8,6) \le 65$, $A_4(11,8)\le 60$ and $A_3(16,11) \le 29$. These in turn imply the new upper bounds $A_5(9,6) \le 325$, $A_5(10,6) \le 1625$, $A_5(11,6) \le 8125$ and $A_4(12,8) \le 240$. Furthermore, we prove that for $\mu ,q \in \mathbb {N}$, there is a 1–1-correspondence between symmetric $(\mu ,q)$-nets (which are certain designs) and codes $C \subseteq [q]^{\mu q}$ of size $\mu q^2$ with minimum distance at least $\mu q - \mu$. We derive the new upper bounds $A_4(9,6) \le 120$ and $A_4(10,6) \le 480$ from these ‘symmetric net’ codes.

## Introduction

For any $$m \in \mathbb {N}$$, we write $$[m]:=\{1,\ldots ,m\}$$. Fix $$n,q \in \mathbb {N}$$. A *word* is an element $$v \in [q]^n$$. So [*q*] serves as the alphabet. (If you prefer $$\{0,1,\ldots ,q-1\}$$ as alphabet, take the letters mod *q*.) For two words $$u,v \in [q]^n$$, their *(Hamming) distance* $$d_H(u,v)$$ is the number of indices *i* with $$u_i \ne v_i$$. A *code* is a subset of $$ [q]^n$$. For any code $$C \subseteq [q]^n$$, the minimum distance $$d_{\text {min}}(C)$$ of *C* is the minimum distance between any two distinct code words in *C*. For $$d \in \mathbb {N}$$, an $$(n,d)_q$$ *-code* is a set $$C\subseteq [q]^n$$ that satisfies $$d_{\text {min}}(C)\ge d$$. Define

1 $$\begin{aligned} A_q(n,d):= \max \{ | C| \,\, | \,\, C \text { is an } (n,d)_q-\hbox {code} \}. \end{aligned}$$

Computing $A_q(n,d)$ and finding upper and lower bounds for it is a long-standing research interest in combinatorial coding theory (cf. MacWilliams and Sloane [12]). In this paper we find new upper bounds on $A_q(n,d)$ (for some *q*, *n*, *d*), based on a *divisibility*-argument. In some cases, it will sharpen a combination of the following two well-known upper bounds on $A_q(n,d)$. Fix $q,n,d \in \mathbb {N}$. Then

2 $$\begin{aligned} qd >(q-1)n\,\,\, \Longrightarrow \,\,\, A_q(n,d) \le \frac{qd}{qd-n(q-1)}. \end{aligned}$$

This is the *q*-ary *Plotkin* bound. Moreover,

3 $$\begin{aligned} A_q(n,d) \le q \cdot A_q(n-1,d). \end{aligned}$$

A proof of these statements can be found in [12]. Plotkin’s bound can be proved by comparing the leftmost and rightmost terms in (4) below. The second bound follows from the observation that in a $(n,d)_q$-code any symbol can occur at most $A_q(n-1,d)$ times at the first position.

We view an $(n,d)_q$-code *C* of size *M* as an $M \times n$ matrix with the words as rows. Two codes $C,D \subseteq [q]^n$ are *equivalent* (or *isomorphic*) if *D* can be obtained from *C* by first permuting the *n* columns of *C* and subsequently applying to each column a permutation of the *q* symbols in [*q*] (we will write ‘*renumbering a column*’ instead of ‘applying a permutation to the symbols in a column’).

If an $(n,d)_q$-code *C* is given, then for $j=1,\ldots ,n$, let $c_{\alpha ,j}$ denote the number of times symbol $\alpha \in [q]$ appears in column *j* of *C*. For any two words $u,v \in [q]^n$, we define $g(u,v):=n-d_H(u,v)$. In our divisibility arguments, we will use the following observations (which are well known and often used in coding theory and combinatorics).

### Proposition 1.1

If *C* is an $(n,d)_q$-code of size *M*, then

4 $$\begin{aligned} \left( {\begin{array}{c}M\\ 2\end{array}}\right) (n-d) \ge \sum _{\begin{array}{c} \{u,v\} \subseteq C\\ u \ne v \end{array}} g(u,v) = \sum _{j=1}^n \sum _{\alpha \in [q]} \left( {\begin{array}{c}c_{\alpha ,j}\\ 2\end{array}}\right) \ge n \cdot \left( (q-r)\left( {\begin{array}{c}m\\ 2\end{array}}\right) +r \left( {\begin{array}{c}m-1\\ 2\end{array}}\right) \right) , \end{aligned}$$

where $$m:= \lceil {M/q}\rceil $$ and $$ r:=qm-M$$, so that $$M=qm-r$$ and $$0 \le r < q$$. Moreover, writing *L* and *R* for the leftmost term and the rightmost term in (4), respectively, we have

5 $$\begin{aligned} | \{ \{u,v\} \subseteq C \,\, | \,\, u \ne v, \,\, d_H(u,v)\ne d \}| \le L - R, \end{aligned}$$

i.e., the number of pairs of distinct words $\{u,v\} \subseteq C$ with distance *unequal* to *d* is at most the leftmost term minus the rightmost term in (4).

**Table 1:** An overview of the results obtained and discussed in this paper

$A_q(n,d)$	Lower bound	Upper bound	New upper bound
	[4, 6, 11]	[4, 6]	
$A_5(8,6)$	50	75	65
$A_5(9,6)$	135	375	325
$A_5(10,6)$	625	1855	1625
$A_5(11,6)$	3125	8840	8125
$A_4(9,6)$	64	128	120
$A_4(10,6)$	256	496	480
$A_4(11,8)$	48	64	60
$A_4(12,8)$	128	242	240
$A_3(16,11)$	18	30	29

All previous lower and upper bounds are taken from references [4, 6], except for the lower bounds $A_5(8,6)\ge 50$ and $A_4(11,8) \ge 48$. These follow from the exact values $A_5(10,8)=50$ and $A_4(12,9)=48$ ([11]). For updated tables with all most recent code bounds, we refer to [7]

### Proof

The first inequality in (4) holds because $n-d \ge g(u,v)$ for all $u,v \in C$. The equality is obtained by counting the number of equal pairs of entries in the same columns of *C* in two ways. The second inequality follows from the (strict) convexity of the binomial coefficient $F(x):=x(x-1)/2$. Fixing a column *j*, the quantity $\sum _{\alpha \in [q]} F(c_{\alpha ,j})$, under the condition that $\sum _{\alpha \in [q]} c_{\alpha ,j} = M$, is minimal if the $c_{\alpha ,j}$ are as equally divided as possible, i.e., if $c_{\alpha ,j} \in \{ \lceil {M/q}\rceil , \lfloor {M/q}\rfloor \}$ for all $\alpha \in [q]$. The desired inequality follows.

To prove the second assertion, note that it follows from (4) that $\sum _{\{u,v\} \subseteq C, \, u \ne v} g(u,v) \ge R$, so

6 $$\begin{aligned} | \{ \{u,v\} \subseteq C \,\, | \,\, u \ne v, \,\, d_H(u,v)\ne d \}|&\le \sum _{\begin{array}{c} \{u,v\} \subseteq C\\ u \ne v \end{array}} (n-d-g(u,v))\nonumber \\&\le \left( {\begin{array}{c}M\\ 2\end{array}}\right) (n-d) - R = L-R. \end{aligned}$$

$$\square $$

### Corollary 1.2

If, for some *q*, *n*, *d* and *M*, the left hand side equals the right hand side in (4), then for any $(n,d)_q$-code *C* of size *M*,

(i) $g(u,v)=n-d$ for all $u,v \in C$ with $u \ne v$, i.e., *C* is *equidistant*, and
(ii) for each column $C_j$ of *C*, there are $q-r$ symbols in [*q*] that occur *m* times in $C_j$ and *r* symbols in [*q*] that occur $m-1$ times in $C_j$.

In the next sections we will use (i), (ii) and the bound in (5) to give (for some *q*, *n*, *d*) new upper bounds on $A_q(n,d)$, based on divisibility arguments. Furthermore, in Sect. 5, we will prove that, for $\mu ,q \in \mathbb {N}$, there is a 1–1-correspondence between symmetric $(\mu ,q)$-nets (which are certain designs) and $(n,d)_q=(\mu q, \mu q - \mu )_q$-codes *C* with $|C|=\mu q^2$. We derive some new upper bounds from these ‘symmetric net’ codes. See Table 1 for an overview of the obtained new upper bounds on $A_q(n,d)$.

## The divisibility argument

In this section, we describe the divisibility argument and illustrate it by an example. Next, we show how the divisibility argument can be applied to obtain upper bounds on $A_q(n,d)$ for certain *q*, *n*, *d*. In subsequent sections, we will see how we can improve upon these bounds for certain fixed *q*, *n*, *d*. We will use the following notation.

### Definition 2.1

($k-$ *block*) Let *C* be an $(n,d)_q$-code in which a symbol $\alpha \in [q]$ is contained exactly *k* times in column *j*. The $k \times n$ matrix *B* formed by the *k* rows of *C* that have symbol $\alpha$ in column *j* is called a (*k*-)*block* (for column *j*). In that case, columns $[n]\setminus \{j\}$ of *B* form an $(n-1,d)_q$-code of size *k*.

At the heart of the divisibility arguments that will be used throughout this paper lies the following observation.

### Proposition 2.2

(Divisibility argument) Suppose that *C* is an $(n,d)_q$-code and that *B* is a block in *C* (for some column *j*) containing every symbol exactly *m* times in every column except for column *j*. If $n-d$ does not divide $m(n-1)$, then for each $u \in C \setminus B$ there is a word $v \in B$ with $d_H(u,v) \notin \{d,n\}$.

### Proof

Let $u \in C \setminus B$. We renumber the symbols in each column such that *u* is $\mathbf {1}:=1\ldots 1$, the all-ones word. The total number of 1’s in *B* is $m(n-1)$ (as the block *B* does not contain 1’s in column *j* since $u\notin B$ and *B* consists of all words in *C* that have the same symbol in column *j*). Since $n-d$ does not divide $m(n-1)$, there must be a word $v \in B$ that contains a number of 1’s not divisible by $n-d$. In particular, the number of 1’s in *v* is different from 0 and $n-d$. So $d_H(u,v) \notin \{d,n\}$. $\square$

### Example 2.3

We apply Proposition 2.2 to the case $(n,d)_q=(8,6)_5$. The best known upper bound^1^ is $A_5(8,6) \le 75$, which can be derived from (2) and (3), as the Plotkin bound yields $A_5(7,6) \le 15$ and hence $A_5(8,6) \le 5 \cdot 15 = 75$. Since, for $(n,d)_q=(7,6)_5$ and $M=15$, the left hand side equals the right hand side in (4), any $(7,6)_5$-code *D* of size 15 is equidistant and each symbol appears exactly $m=3$ times in every column of *D*. Note $2=n-d \not \mid m(n-1)=21$.

Suppose there exists a $(8,6)_5$-code *C* of size 75. As $A_5(7,6) \le 15$, for each column, *C* is divided into five 15-blocks. Let *B* be a 15-block for the *j*th column and let $u \in C \setminus B$. Note that the other columns of *B* contain each symbol 3 times, and $3(n-1)=3\cdot 7 =21$ is not divisible by $n-d=2$. So by Proposition 2.2, there must be a word $v \in B$ with $d_H(u,v) \notin \{6,8\}$.

However, since all $(7,6)_5$-codes of size 15 are equidistant, all distances in *C* belong to $\{6,8\}$: either two words are contained together in some 15-block (hence their distance is 6) or there is no column for which the two words are contained in a 15-block (hence their distance is 8). This implies that an $(8,6)_5$-code *C* of size 75 cannot exist. Hence $A_5(8,6) \le 74$. Theorem 2.5 and Corollary 2.6 below will imply that $A_5(8,6)\le 70$ and in Sect. 3 we will show that, with some computer assistance, the bound can be pushed down to $A_5(8,6) \le 65$.

To exploit the idea of Proposition 2.2, we will count the number of so-called irregular pairs of words occuring in a code.

### Definition 2.4

*(Irregular pair)* Let *C* be an $(n,d)_q$-code and $u,v \in C$ with $u\ne v$. If $d_H(u,v) \notin \{d,n\}$, we call $\{u,v\}$ an *irregular pair*.

For any code $C \subseteq [q]^n$, we write

7 $$\begin{aligned} X:= \text { the set of irregular pairs }~\{u,v\} \text { for }~u,v \in C. \end{aligned}$$

Using Proposition 2.2, we can for some cases derive a lower bound on |*X*|. If we can also compute an upper bound on |*X*| that is smaller than the lower bound, we derive that the code *C* cannot exist. The proof of the next theorem uses this idea. For fixed $q,n,d,m \in \mathbb {N}$ with $q \ge 2$, define the following quadratic polynomial in *r*:

8 $$\begin{aligned} \phi (r):= n(n-1-d)(r-1)r - (q-r+1)(mq(q+r-2)-2r). \end{aligned}$$

### Theorem 2.5

Suppose that $q\ge 2$, that $m:=d/(qd-(n-1)(q-1))$ is a positive integer, and that $n-d$ does not divide $m(n-1)$. If $r \in \{1,\ldots ,q-1\}$ with $\phi (r)<0$, then $A_q(n,d) < mq^2 -r$.

### Proof

By Plotkin’s bound (2) we have

9 $$\begin{aligned} A_q(n-1,d) \le mq. \end{aligned}$$

Let *D* be an $$(n-1,d)_q$$-code of size $$mq-t$$ with $$t<q$$. Note that $$d=m(n-1)(q-1)/(mq-1)$$. Then the right-hand side in (5) (taking $$C:= D)$$ is equal to $$(n-1)(m-1)t(t-1)/(2mq-2) = (n-1-d)\left( {\begin{array}{c}t\\ 2\end{array}}\right) $$. Hence

10 $$\begin{aligned} D \text { contains at most }~ (n-1-d)\left( {\begin{array}{c}t\\ 2\end{array}}\right) \text { pairs of words with distance }~\ne d. \end{aligned}$$

Therefore, all $(n-1,d)_q$-codes *D* of size *mq* are equidistant (then $t=0$) and each symbol occurs *m* times in every column of *D*.

Now let *C* be an $(n,d)_q$-code of size $M:=mq^2-r$ with $r \in \{1,\ldots ,q-1\}$. Consider an *mq*-block *B* for some column of *C*. As $n-d$ does not divide $m(n-1)$, by Proposition 2.2 we know

11 $$\begin{aligned} \text {if}~u \in C\setminus B, \text { then there exists }~v \in B\,\text {with }~d_H(u,v) \notin \{d,n\}. \end{aligned}$$

Let $B_1,\ldots , B_{s}$ be *mq*-blocks in *C* for some fixed column. Since $|C|=mq^2-r$, the number of *mq*-blocks for any fixed column is at least $q-r$ (so we can take $s=q-r$). Then, with (11), one obtains a lower bound on the number |*X*| of irregular pairs in *C*. Every pair $\{B_{i},B_{k}\}$ of *mq*-blocks gives rise to *mq* irregular pairs: for each word $u \in B_{i}$, there is a word $v \in B_k$ such that $\{u,v\}\in X$. This implies that in $\cup _{i=1}^s B_i \subseteq C$ there are at least $$\left( {\begin{array}{c}s\\ 2\end{array}}\right) mq$$ irregular pairs. Moreover, for each word *u* in $C\setminus \cup _{i=1}^s B_i$ (there are $M-mq\cdot s$ of such words) there is, for each $i=1,\ldots ,s$, a word $v_i \in B_i$ with $\{u,v_i\} \in X$. This gives an additional number of at least $(M-mqs)s$ irregular pairs in *C*. Hence:

12 $$\begin{aligned} |X|&\ge \left( {\begin{array}{c}s\\ 2\end{array}}\right) mq + (M-mqs)s \nonumber \\&= \frac{1}{2}s (mq(2q-s-1)-2r) =: l(s). \end{aligned}$$

On the other hand, note that the *i*th block for the *j*th column has size $mq-r_{i,j}$ for some integer $r_{i,j}\ge 0$ by (9), where $\sum _{i=1}^q r_{i,j}=r\le q-1$ (hence each $r_{i,j} <q$). So by (10), the number of irregular pairs in *C* that have the same entry in column *j* is at most

13 $$\begin{aligned} (n-1-d)\sum _{i=1}^q \left( {\begin{array}{c}r_{i,j}\\ 2\end{array}}\right) . \end{aligned}$$

As each irregular pair $\{u,v\}$ has $u_j=v_j$ for at least one column *j*, we conclude

14 $$\begin{aligned} |X| \le (n-1-d)\sum _{j=1}^n \sum _{i=1}^q \left( {\begin{array}{c}r_{i,j}\\ 2\end{array}}\right) \le n (n-1-d)\left( {\begin{array}{c}r\\ 2\end{array}}\right) . \end{aligned}$$

Here the last inequality follows by convexity of the binomial function, since (for fixed *j*) the sum $$\sum _{i=1}^q \left( {\begin{array}{c}r_{i,j}\\ 2\end{array}}\right) $$ under the condition that $\sum _{i=1}^q r_{i,j}=r$ is maximal if one of the $r_{i,j}$ is equal to *r* and the others are equal to 0.

If each $r_{i,j}\in \{0,1\}$, then $|X|=0$ by (14). As $q-r\ge 1$, there is at least one *mq*-block for any fixed column, so $|X|\ge 1$ by (11), which is not possible. Hence we can assume that $r_{i,j} \ge 2$ for some *i*, *j* (this also implies $A_q(n,d) \le mq^2-2$). Then the number *s* of *mq*-blocks for column *j* satisfies $s \ge q-r+1$. This gives by (12) and (14) that

15 $$\begin{aligned} l(q-r+1) \le |X| \le (n-1-d) \left( {\begin{array}{c}r\\ 2\end{array}}\right) . \end{aligned}$$

Subtracting the left hand side from the right hand side in (15) yields $\phi (r)/2 \ge 0$, i.e., $\phi (r) \ge 0$. So if $\phi (r)< 0$, then $A_q(n,d) < mq^2-r$, as was needed to prove. $\square$

We give two interesting applications of Theorem 2.5.

### Corollary 2.6

If $q \equiv 1 \pmod {4}$ and $q\ne 1$, then

16 $$\begin{aligned} A_q(q+3,q+1) \le \frac{1}{2}q^2(q+1)-q =\frac{1}{2}(q-1)q (q+2). \end{aligned}$$

### Proof

Apply Theorem 2.5 to $n=q+3$, $d=q+1$ and $r=q-1$. Then $m=(q+1)/2 \in \mathbb {N}$ and $n-d=2$ does not divide $m(n-1)=(q+1)(q+2)/2$, as $q\equiv 1 \pmod {4}$. Furthermore, $\phi (q-1)=-(q^3-q^2-2)<0$. Hence $A_q(q+3,q+1) < q^2(q+1)/2-(q-1)$. $\square$

Applying Corollary 2.6 to $q=5$ gives $A_5(8,6) \le 70$. In Sect. 3 we will improve this to $A_5(8,6) \le 65$.

### Remark 2.7

Note that for bound (16) to hold it is necessary that $q\equiv 1 \pmod {4}$. If $q \equiv 3 \pmod {4}$ the statement does *not* hold in general. For example, $A_3(6,4)= 18$ (see [7]), which is larger than bound (16).

Theorem 2.5 also gives an upper bound on $A_q(n,d)=A_q(kq+k+q,kq)$, where $q\ge 2$ and *k* does not divide $q(q+1)$ (which is useful for $k <q-1$; for $k \ge q+1$ the Plotkin bound gives a better bound). One new upper bound for such *q*, *n*, *d* is obtained:

### Proposition 2.8

$$A_4(11,8)\le 60$$.

### Proof

This follows from Theorem 2.5 with $q=4$, $n=11$, $d=8$ and $r=3$. Then $m=4 \in \mathbb {N}$, and $n-d=3$ does not divide $m(n-1)=40$. Moreover, $\phi (3)=-16 <0$. Therefore $A_4(11,8) < 61$. $\square$

This implies the following bound, which is also new:

### Corollary 2.9

$$A_4(12,8) \le 240$$.

### Proof

By Proposition 2.8 and (3). $\square$

## Kirkman triple systems and $A_5(8,6)$.

In this section we consider the case $(n,d)_q=(8,6)_5$ from Example 2.3. Corollary 2.6 implies that $A_5(8,6) \le 70$. Using small computer experiments, we will obtain $A_5(8,6) \le 65$.

As in the proof of Theorem 2.5, we will compare upper and lower bounds on |*X*|. But since an $(8,6)_5$-code *C* of size at most 70 does not necessarily contain a 15-block (as $70=5\cdot 14$), we need information about 14-blocks. To this end we show, using an analogous approach as in [5] (based on occurrences of symbols in columns of an equidistant code):

### Proposition 3.1

Any $(7,6)_5$-code *C* of size 14 can be extended to a $(7,6)_5$-code of size 15.

### Proof

For $M=14$, the leftmost term in (4) equals the rightmost term. So *C* is equidistant and for each $j \in \{1,\ldots ,7\}$ there exists a unique $\beta _j \in [q]$ with $c_{\beta _j,j} =2$ and $c_{\alpha ,j}=3$ for all $\alpha \in [q] \setminus \{\beta _j \}$. We can define a 15-th codeword *u* by putting $u_j:= \beta _j$ for all $j=1,\ldots ,7$. We claim that $C \cup \{ u \}$ is a $(7,6)_5$-code of size 15.

To establish the claim we must prove that $d_H(u,w) \ge 6$ for all $w \in C$. Suppose that there is a word $w \in C$ with $d_H(u,w) <6$. We can renumber the symbols in each column of *C* such that $w=\mathbf {1}$. Since *C* is equidistant, each word in $C \setminus \{ w \}$ contains precisely one 1. On the other hand, there are two column indices $j_1$ and $j_2$ with $u_{j_1} = 1$ and $u_{j_2} = 1$. Then $C\setminus \{w\}$ contains at most $1+1+5\cdot 2 = 12$ occurrences of the symbol 1 (since in columns $j_1$ and $j_2$ there is precisely one 1 in $C \setminus \{w\}$). But in that case, since $|C\setminus \{ w \}|=13>12$, there is a row in *C* that contains zero occurrences of the symbol 1, contradicting the fact that *C* is equidistant. $\square$

Note that a code of size more than 65 must have at least one 15- or 14-block, and therefore it must have a subcode of size 65 containing at least one 15- or 14-block. We shall now prove that this is impossible because

17 $$\begin{aligned} \text {each}~(8,6)_5\text {-code of size }~65 \text { only admits }~13\text {-blocks.} \end{aligned}$$

It follows that $A_5(8,6) \le 65$. In order to prove (17), let *C* be a $(8,6)_5$-code of size 65. We first compute a lower bound on the number of irregular pairs in *C*. Define, for $x,y \in \mathbb {Z}_{\ge 0}$,

18 $$\begin{aligned} f(x,y)&:= (3 x+ y)(65-15 x-14 y) + 3 \cdot 15 \left( {\begin{array}{c}x\\ 2\end{array}}\right) + 14 \left( {\begin{array}{c}y\\ 2\end{array}}\right) + 3\cdot 14 xy\nonumber \\&\phantom {={,}} - 2 \cdot 21 x- 8 y + \mathbf {1}_{\{y>0 \text { and } x=0\}} (65-14-39). \end{aligned}$$

### Proposition 3.2

(Lower bound on |*X*|) Let *C* be an $(n,d)_q=(8,6)_5$-code of size 65 and let $j \in [n]$. Let *x* and *y* be the number of symbols that appear 15 and 14 times (respectively) in column *j*. Then the number |*X*| of irregular pairs in *C* is at least *f*(*x*, *y*).

### Proof

First consider a $(7,6)_5$-code *D* of size 15 or size 14 and define

19 $$\begin{aligned} S:= \{ u \in [5]^7 \,\, | \,\,d_H(w,u) \ge 5 \,\,\,\, \forall \, w \in D \}. \end{aligned}$$

For any $u \in S$, define

20 $$\begin{aligned} \alpha (u):= |\{w \in D\,\,: \, \, d_H(u,w)=6 \}|. \end{aligned}$$

Then

21 $$\begin{aligned} \text {if } |D|=15, \text { then} \phantom {aaai}&\text {if } |D|=14, \text { then}\phantom {aaai}&\nonumber \\ | \{u \in S\,\, |\,\, \alpha (u)=0\}|&=0,&| \{u \in S\,\, |\,\, \alpha (u)=0\}|&\le 8, \nonumber \\ | \{u \in S\,\, |\,\, \alpha (u)=1\}|&\le 21,&|\{ u \in S\,\, |\,\, \alpha (u)\le 1\}|&\le 39. \nonumber \\ | \{u \in S\,\, |\,\, \alpha (u)=2\}|&=0.&\end{aligned}$$

This can be checked efficiently with a computer^2^ by checking all possible $(7,6)_5$-codes of size 15 and 14 up to equivalence. Here we note that a $(7,6)_5$-code *D* (which must be equidistant, see Example 2.3) of size 15 corresponds to a solution to *Kirkman’s school girl problem* [16].^3^ So to establish (21), it suffices to check^4^ all $(7,6)_5$-codes of size 15, that is, Kirkman systems (there are 7 nonisomorphic Kirkman systems [8]), and all $(7,6)_5$-codes of size 14, of which there are at most $7 \cdot 15$ by Proposition 3.1.

Let $G=(C,X)$ be the graph with vertex set $V(G):=C$ and edge set $E(G):=X$. Consider a 15-block *B* determined by column *j*. By (21), each $u \in C \setminus B$ has $\ge 1$ neighbour in *B*. We observed this also in Example 2.3: for any $u \in C \setminus B$ there exists at least one $v \in B$ such that $d_H(u,v) \notin \{6,8\}$, so $d_H(u,v)=7$ and $\{u,v\} \in X$. In (21) this is represented as: if $|D|=15$ then $|\{u \in S\,\, | \,\, \alpha (u)=0\}|=0$, i.e., for any word $u'$ of length 7 that has distance $\ge 5$ to all words in a $(7,6)_5$-code *D* of size 15, there is at least one $v'\in D$ such that $d_H(u',v')=6$.

Furthermore, (21) gives that all but $\le 21$ elements $u\in C\setminus B$ have $\ge 3$ neighbours in *B*. So by adding $\le 2 \cdot 21$ new edges, we obtain that each $u \in C \setminus B$ has $\ge 3$ neighbours in *B*.

Similarly, for any 14-block *B* determined by column *j*, by adding $\le 8$ new edges we achieve that each $u \in C \setminus B$ has $\ge 1$ neighbour in *B*. Hence, by adding $\le ( 2 \cdot 21 \cdot x+ 8\cdot y )$ edges to *G*, we obtain a graph $G'$ with

22 $$\begin{aligned} |E(G')| \ge (3 x+ y)(65-15 x-14 y) + 3 \cdot 15 \left( {\begin{array}{c}x\\ 2\end{array}}\right) + 14 \left( {\begin{array}{c}y\\ 2\end{array}}\right) + 3\cdot 14 xy. \end{aligned}$$

This results in the required bound, except for the term with the indicator function. That term can be added because $|\{ u \in S\,\, |\,\, \alpha (u)\le 1\}| \le 39$ if $|D|=14$, by (21). $\square$

It is also possible to give an upper bound on |*X*|. If *D* is a $(7,6)_5$-code of size *k*, an upper bound $h(k)=L-R$ on the number of pairs $\{u,v\} \subseteq D$ with $u \ne v$ and $d_H(u,v)\ne 6$ (hence $d_H(u,v)=7$) is given by (5). The resulting values *h*(*k*) are given in Table 2.

**Table 2:** Upper bound *h*(*k*) on the number of pairs $\{u,v\} \subseteq D$ with $d_H(u,v)=7$ for a $(7,6)_5$-code *D* with $|D|=k$

*k*	15	14	13	12	11	10	9	8	7	6	5
*h*(*k*)	0	0	1	3	6	10	8	7	7	8	10

### Theorem 3.3

($A_{5}(8,6) \le 65$) Suppose that *C* is an $(n,d)_q=(8,6)_5$-code with $|C|=65$. Then each symbol appears exactly 13 times in each column of *C*. Hence, $A_{5}(8,6) \le 65$.

### Proof

Let $a^{(j)}_{k}$ be the number of symbols that appear exactly *k* times in column *j* of *C*. Then the number of irregular pairs that have the same entry in column *j* is at most $\sum _{k =5}^{15} a^{(j)}_{k}h(k)$. It follows that

23 $$\begin{aligned} |X| \le U := \sum _{j=1}^8 \sum _{k =5}^{15} a^{(j)}_{k} h(k). \end{aligned}$$

One may check that if $\mathbf {a}, \mathbf {b} \in \mathbb {Z}_{\ge 0}^{15}$ are 15-tuples of nonnegative integers, with $\sum _k a_k k =65$, $\sum _k b_k k =65$, $\sum _k a_k=5$, $\sum _k b_k=5$, and $f(a_{15},a_{14}) \le f(b_{15},b_{14}) \ne 0$, then

24 $$\begin{aligned} \sum _{k=5}^{15} (7a_k +b_k) h(k) < f(b_{15},b_{14}). \end{aligned}$$

[There are 30 $\mathbf {a} \in \mathbb {Z}_{\ge 0}^{15}$ with $\sum _k a_k k =65$ and $\sum _{k}a_k = 5$. So there are 900 possible pairs $\mathbf {a},\mathbf {b}$. A computer now quickly verifies (24)].

By permuting the columns of *C* we may assume that $\max _j f( a^{(j)}_{15},a^{(j)}_{14})=f(a^{(1)}_{15},a^{(1)}_{14})$. Hence if $f(a^{(1)}_{15},a^{(1)}_{14})>0$, then

25 $$\begin{aligned} U&= \sum _{j=1}^8 \sum _{k =5}^{15} a^{(j)}_{k} h(k) = \frac{1}{7}\sum _{j=2}^8\left( \sum _{k =5}^{15} \left( 7a^{(j)}_{k} + a^{(1)}_{k} \right) h(k) \right) \nonumber \\&< f\left( a^{(1)}_{15},a^{(1)}_{14}\right) \le |X| \end{aligned}$$

(where we used Proposition 3.2 in the last inequality), contradicting (23). So $f( a^{(j)}_{15},a^{(j)}_{14})=0$ for all *j*, which implies (for $\mathbf {a}^{(j)} \in \mathbb {Z}_{\ge 0}^{15}$ with $\sum _k a^{(j)}_k k =65$, $\sum _k a^{(j)}_k=5$) that $a^{(j)}_{15}=a^{(j)}_{14}=0$ for all *j*, hence each symbol appears exactly 13 times in each column of *C*. $\square$

### Corollary 3.4

$$A_5(9,6) \le 325$$, $$A_5(10,6) \le 1625$$ and $$A_5(11,6) \le 8125$$.

### Proof

By Theorem 3.3 and (3). $\square$

## Improved bound on $A_3(16,11)$.

We show that $A_3(16,11) \le 29$ using a surprisingly simple argument.

### Proposition 4.1

$$A_3(16,11) \le 29$$.

### Proof

Suppose that *C* is an $(n,d)_q=(16,11)_3$-code of size 30. We can assume that $\mathbf {1} \in C$. It is known that $A_3(15,11)=10$, so the symbol 1 is contained at most 10 times in every column of *C*. Since $|C|=30$, the symbol 1 appears exactly 10 times in every column of *C*, so the number of 1’s in *C* is divisible by 5. On the other hand it is easy to check that a $(15,11)_3$-code of size 10 is equidistant (using (5), as $L=R$). This implies that all distances in a $(16,11)_3$-code of size 30 belong to $\{11, 16\}$. So the number of 1’s in any code word $\ne \mathbf {1}$ is 0 or 5. As $\mathbf {1}$ contains 16 1’s, it follows that the total number of 1’s is not divisible by 5, a contradiction. $\square$

## Codes from symmetric nets

In this section we will show that there is a 1-1-correspondence between *symmetric* $(\mu ,q)$ *-nets* and $(n,d)_q=(\mu q,\mu q-\mu )_q$-codes of size $\mu q^2$. From this, we derive in Sect. 6 the new upper bound $A_4(9,6) \le 120$, implying $A_4(10,6) \le 480$.

### Definition 5.1

*(Symmetric net)* Let $\mu ,q \in \mathbb {N}$. A *symmetric* $(\mu , q)$ *-net* (also called *symmetric transversal design* [3]) is a set *X* of $\mu q^2$ elements, called *points*, together with a collection $\mathcal {B}$ of subsets of *X* of size $\mu q$, called *blocks*, such that:

1. $\mathcal {B}$ can be partitioned into $\mu q$ partitions (*block parallel classes*) of *X*.
2. Any two blocks that belong to different parallel classes intersect in exactly $\mu$ points.
3. *X* can be partitioned into $\mu q$ sets of *q* points (*point parallel classes*), such that any two points from different classes occur together in exactly $\mu$ blocks, while any two points from the same class do not occur together in any block.^5^

### Remark 5.2

From the 1–1-correspondence between symmetric $(\mu ,q)$-nets and $(n,d)_q=(\mu q,\mu q - \mu )_q$-codes *C* of size $\mu q^2$ in Theorem 5.6 below it follows that (s2) and (s3) can be replaced by the single condition:

(s’): Each pair of points is contained in at most $\mu$ blocks,

since the only condition posed on such a code is that $g(u,v) \le \mu$ for all distinct $u,v \in C$.

### Example 5.3

Let $X=\{1,2,3,4\}$ and $\mathcal {B}=\{\{1,3\},\,\{2,4\},\,\{1,4\},\,\{2,3\} \}$. Then $(X,\mathcal {B})$ is a symmetric (1, 2)-net. The block parallel classes are $\{\{1,3\},\,\{2,4\}\}$ and $\{\{1,4\},\,\{2,3\}\}$. The point parallel classes are $\{1,2\}$ and $\{3,4\}$.

By labeling the points as $x_1,\ldots ,x_{\mu q^2}$ and the blocks as $B_1,\ldots ,B_{\mu q^2}$, the $\mu q^2 \times \mu q^2$ *-incidence matrix* *N* of a symmetric $(\mu ,q)$-net is defined by

26 $$\begin{aligned} N_{i,j}:= {\left\{ \begin{array}{ll}1 &{}\text{ if } x_i \in B_j, \\ 0 &{}\text{ else }. \end{array}\right. } \end{aligned}$$

An *isomorphism* of symmetric nets is a bijection from one symmetric net to another symmetric net that maps the blocks of the first net into the blocks of the second net. That is, two symmetric nets are isomorphic if and only if their incidence matrices are the same up to row and column permutations. Symmetric nets are, in some sense, a generalization of *generalized Hadamard matrices*.

### Definition 5.4

*(Generalized Hadamard matrix)* Let *M* be an $n \times n$-matrix with entries from a finite group *G*. Then *M* is called a *generalized Hadamard matrix* GH(*n*, *G*) (or GH(*n*, |*G*|)) if for any two different rows *i* and *k*, the *n*-tuple $(M_{ij}M_{jk}^{-1})_{j=1}^n$ contains each element of *G* exactly *n* / |*G*| times.

**Fig. 1:**
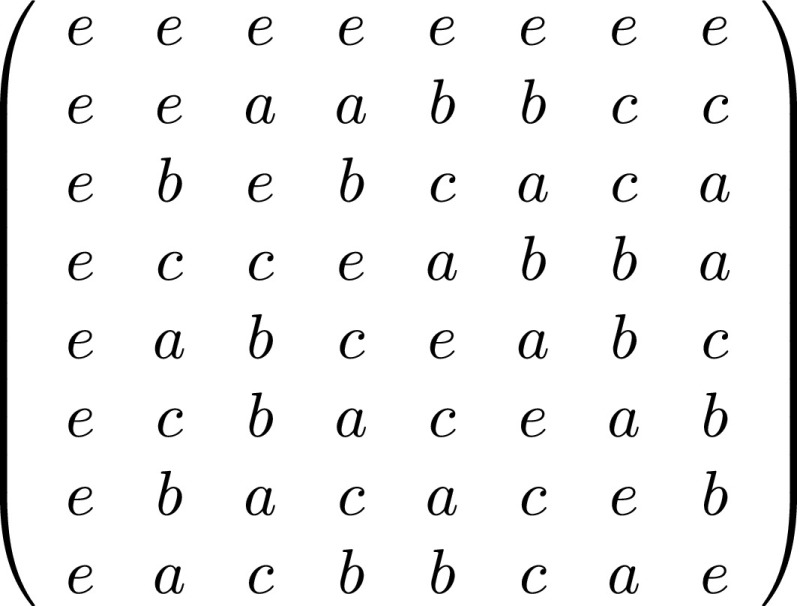
An incidence matrix of the unique (up to isomorphism) symmetric (2, 4)-net is obtained by writing the elements *e*, *a*, *b*, *c* as $4 \times 4$-permutation matrices in the generalized Hadamard matrix GH$(8,V_4)$ (with $V_4$ the Klein 4-group). See Al-Kenani [1]

Each generalized Hadamard matrix GH(*n*, *G*) gives rise to a symmetric (*n* / |*G*|, |*G*|)-net: by replacing *G* by a set of $|G| \times |G|$-permutation matrices isomorphic to *G* (as a group), one obtains the incidence matrix of a symmetric net. See Fig. 1 for an example. Not every symmetric (*n* / *q*, *q*)-net gives rise to a generalized Hadamard matrix GH(*n*, *q*), see [13]. But if the group of automorphisms (*bitranslations*) of a symmetric (*n* / *q*, *q*)-net has order *q*, then one can construct a generalized Hadamard matrix GH(*n*, *q*) from it. See [3] for details.

### Assumption 5.5

In this section we consider triples $(n,d)_q$ of natural numbers for which

27 $$\begin{aligned} qd = (q-1)n, \end{aligned}$$

hence $n-d=n/q=:\mu$ and $\mu \in \mathbb {N}$. So $(n,d)_q=(\mu q, \mu q - \mu )_q$.

The fact that a generalized Hadamard matrix $\text {GH}(n,q)$ gives rise to an $(n,d)_q$-code of size *qn*, was proved in [11] and for some parameters it can also be deduced from an earlier paper [15]. Using a result by Bassalygo et al. [2] about the structure of $(n,d)_q$-codes of size *qn*,^6^ we prove that such codes are in 1–1-relation with symmetric (*n* / *q*, *q*)-nets.

### Theorem 5.6

Let $\mu , q \in \mathbb {N}$. There is a 1–1-relation between symmetric $(\mu ,q)$-nets (up to isomorphism) and $(n,d)_q=(\mu q,\mu q -\mu )_q$-codes *C* of size $\mu q^2$ (up to equivalence).

### Proof

Given an $(n,d)_q= (\mu q,\mu q -\mu )_q$-code *C* of size $\mu q^2$, we construct a (0, 1)-matrix *M* of order $\mu q^2 \times \mu q^2$ with the following properties:

(I) *M* is a $\mu q^2 \times \mu q^2$ matrix that consists of $q \times q$ blocks $\sigma _{i,j}$ (so *M* is a $\mu q \times \mu q$ matrix of blocks $\sigma _{i,j}$), where each $\sigma _{i,j}$ is a permutation matrix.
(II) $MM^T= M^TM= A$, where *A* is a $\mu q^2 \times \mu q^2$ matrix that consists of $q \times q$ blocks $A_{i,j}$ (so *A* is an $\mu q \times \mu q$ matrix of blocks $A_{i,j}$), with
  28 $$\begin{aligned} A_{i,j} ={\left\{ \begin{array}{ll} \mu q \cdot I_q &{}\text{ if } i =j, \\ \mu \cdot J_q &{} \text{ if } i \ne j. \end{array}\right. } \end{aligned}$$
  Here $J_q$ denotes the $q \times q$ all-ones matrix.

By Proposition 4 of [2], since $d=n(q-1)/q$ and $|C|=qn$, *C* can be partitioned as

29 $$\begin{aligned} C = V_1 \cup V_2 \cup \ldots \cup V_{n}, \end{aligned}$$

where the union is disjoint, $|V_i|=q$ for all $i=1,\ldots ,n$, and where $d_H(u,v)=n$ if $u,v \in C$ are together in one of the $V_i$, and $d_H(u,v)=d$ if $u\in V_i$ and $v \in V_j$ with $i\ne j$.

Now we write each word $w \in [q]^{n}$ as a (0, 1)-row vector of size $qn = \mu q^2$ by putting a 1 on positions $(i,w_i) \in [n] \times [q]$ (for $i=1,\ldots ,n$) and 0’s elsewhere. The *q* words in any of the $V_i$ then form a $q \times qn$ matrix consisting of *n* permutation matrices $\sigma _{i,j}$ of size $q \times q$.

By placing the matrices obtained in this way from all *n* tuples $V_1,\ldots ,V_{n}$ underneath each other, we obtain a $qn \times qn$ matrix *M* consisting of $n^2$ permutation matrices of order $q\times q$, so (I) is satisfied. Property (II) also holds, since for any $u,v \in C$ written as row vectors of size *qn*, with the $V_i$ as in (29), it holds that

30 $$\begin{aligned} \sum _{k \in [n] \times [q]}u_kv_k= g(u,v)={\left\{ \begin{array}{ll}n = \mu q &{}\text{ if } u = v, \\ 0 &{}\text{ if } u \ne v \text { and } u,v \in V_i,\\ n-d=\mu &{}\text{ if } u \ne v \text { and } u \in V_i, v \in V_j \text { with } i \ne j.\\ \end{array}\right. } \end{aligned}$$

**Fig. 2:**
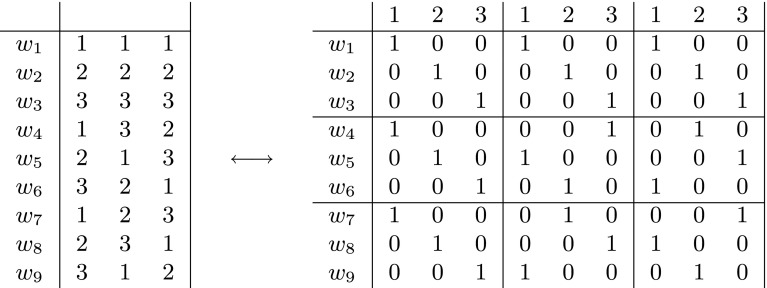
An $(n,d)_q=(3,2)_3$-code $C=\{w_1,\ldots ,w_9\}$ of size 9 (*left* table) gives rise to an incidence matrix of a symmetric (1, 3)-net (*right* table) and vice versa

So $MM^T=A$. Moreover, if $j_1:=(j_1',a_1) \in [n] \times [q]$ and $j_2:=(j_2',a_2) \in [n] \times [q]$, then

31 $$\begin{aligned} \sum _{k \in [qn]}M_{k,j_1} M_{k,j_2}={\left\{ \begin{array}{ll}n = \mu q &{}\text{ if } j_1'=j_2' \text { and } a_1=a_2, \\ 0 &{}\text{ if } j_1' = j_2' \text { and } a_1\ne a_2,\\ n/q = \mu &{}\text{ if } j_1' \ne j_2',\\ \end{array}\right. } \end{aligned}$$

where the last statement follows by considering the words in *C* that have $a_1$ at the $j_1'$-th position. (The remaining columns form an *n*-block for the $j_1'$-th column. In this *n*-block, each symbol occurs exactly *n* / *q* times at each position, since the leftmost term equals the rightmost term in (4) for $(n-1,d)_q$-codes of size *n*.) We see that also $M^TM=A$. Hence, *M* is the incidence matrix of a symmetric $(\mu ,q)$-net (see [3, Proposition I.7.6] for the net and its dual). See Fig. 2 for an example.

Note that one can do the reverse construction as well: given a symmetric $(\mu ,q)$-net, the incidence matrix of *M* can be written (after possible row and column permutations) as a matrix of permutation matrices such that $MM^T=M^TM=A$, with *A* as in (28). From *M* we obtain a code *C* of size $\mu q^2$ of the required minimum distance by mapping the rows $(i,w_i) \in [\mu q] \times [q]$ to $w \in [q]^{\mu q}$. Observe that equivalent codes yield isomorphic incidence matrices *M* and vice versa. $\square$

## New upper bound on $A_4(9,6)$.

In this section we use the 1–1-correspondence between symmetric $(\mu ,q)$-nets and $(n,d)_q=(\mu q, \mu q - \mu )_q$-codes of size $\mu q^2$ in combination with a known result about symmetric (2, 4)-nets [1] to derive that $A_4(9,6) \le 120$.

As $A_4(8,6)=32$, any $(9,6)_4$-code of size more than 120 must contain at least one 31- or 32-block, and therefore it contains a subcode of size 120 containing at least one 31- or 32-block. We will show (using a small computer check) that this is impossible because a $(9,6)_4$-code of size 120 does not contain any 31- or 32-blocks. Therefore $A_4(9,6) \le 120$. In order to do prove this, we need information about $(8,6)_4$-codes of size 31.

### Proposition 6.1

Let $q,n,d \in \mathbb {N}$ satisfy $qd=(q-1)n$. Any $(n,d)_q$-code *C* of size $qn-1$ can be extended to an $(n,d)_q$-code of size *qn*.

### Proof

Let *C* be an $(n,d)_q$-code of size $qn-1$. By Plotkin’s bound, $A_q(n-1,d) \le n$, so each symbol occurs at most *n* times in each column of *C*, hence there exists for each $j \in [n]$ a unique $\beta _j \in [q]$ with $c_{\beta _j,j} =n-1$ and $c_{\alpha ,j}=n$ for all $\alpha \in [q] \setminus \{\beta _j \}$. We can define a *qn*-th codeword *u* by putting $u_j:= \beta _j$ for all $j=1,\ldots ,n$. We claim that $C \cup \{ u \}$ is an $(n,d)_q$-code of size *qn*.

To establish the claim we must prove that $d_H(u,w) \ge d$ for all $w \in C$. Let $w \in C$ with $d_H(u,w) < n$. We can renumber the symbols in each column of *C* such that $w=\mathbf {1}$. Then *w* is contained in an $(n-1)$-block *B* for some column in *C* (otherwise $d_H(u,w)=n$). The number of 1’s in *B* is $n+(n-2)n/q$ (since any $(q,n-1,d)$-code of size $n-1$ is equidistant, as $L-R=0$ in (5) for $(n-1,d)_q$-codes of size $n-1$) and the number of 1’s in $C \setminus B$ is $(q-1)(n-1)n/q$ (since in any $(n-1,d)_q$-code of size *n*, each symbol appears exactly *n* / *q* times in each column, as the leftmost term equals the rightmost term in (4) for $(n-1,d)_q$-codes of size *n*). Adding these two numbers we see that the number of 1’s in *C* is $n^2-n/q$. Since $C \cup \{u\}$ contains each symbol $n^2$ times by construction, *u* contains symbol 1 exactly *n* / *q* times, hence $d_H(u,w)=n-n/q=d$, which gives the desired result. $\square$

### Proposition 6.2

$$A_4(9,6) \le 120$$.

### Proof

The $(n,d)_q=(8,6)_4$-code of size 32 is unique up to equivalence, since the symmetric (2, 4)-net is unique up to equivalence (see Al-Kenani [1]). By checking all $(8,6)_4$-codes *D* of size 31 (of which there are at most 32 up to equivalence since each $(8,6)_4$-code of size 31 arises by removing one word from a $(8,6)_4$-code of size 32 by Proposition 6.1) we find that

32 $$\begin{aligned} |\{ u \in [4]^{8} \,\, | \,\,d_H(w,u) \ge 5 \,\,\,\, \forall \, w \in D \}| \le 25. \end{aligned}$$

This implies that an $(n,d)_q=(9,6)_4$-code *C* of size 120 cannot contain a 31- or 32-block. Therefore $A_4(9,6) \le 120$. $\square$

### Corollary 6.3

$$A_4(10,6) \le 480$$.

### Proof

By Proposition 6.2 and (3). $\square$

## Footnotes

1 The Delsarte bound [9] on $A_5(8,6)$, the bound based on Theorem 2, and the semidefinite programming bound based on quadruples of code words [10] all are equal to 75.

2 All computer tests in this paper are small and can be executed within a minute on modern personal computers.

3 Kirkman’s school girl problem asks to arrange 15 girls 7 days in a row in groups of 3 such that no two girls appear in the same group twice. The 1–1-correspondence between $(n,d)_q=(7,6)_5$-codes *D* of size 15 and solutions to Kirkman’s school girl problem is given by the rule: girls  $i_1$ and  $i_2$ walk in the same triple on day  *j* $\Longleftrightarrow D_{i_1,j} = D_{i_2,j}$.

4 By ‘check’ we mean that given a $(7,6)_5$-code *D* of size 14 or 15, we first compute *S*, then $\alpha (u)$ for all $u \in S$, and subsequently verify (21).

5 That is, a symmetric $(\mu ,q)$-net is a $1-(\mu q^2, \mu q, \mu q)$ design *D*, which is resolvable (s1), affine (s2), and the dual design $D^*$ of *D* is affine resolvable (s3).

6 Note that $A_q(n,d)\le qn$, since by Plotkin’s bound (2), $A_q(n-1,d) \le n$, hence $A_q(n,d) \le qn =\mu q^2$ by (3).
